# Laparoscopic partial nephrectomy for multiple (four) tumors

**DOI:** 10.1590/S1677-5538.IBJU.2015.0648

**Published:** 2017

**Authors:** Lessandro Curcio, Bruno Salama, Daniel Luis Pinto, Antonio Claudio Ahouagi

**Affiliations:** 1 Hospital Federal Ipanema, RJ, Brasil

## Abstract

**Background:**

Nephron sparing surgery (NSS) is well established as the standard of care for most surgical small renal tumors when technically feasible. While the majority of sporadic renal tumors are solitary, multifocal tumors have been reported in 5.4% to 25% of patients with tumors smaller than 5cm. We present a video where we approach, through laparoscopy, four tumors on the same kidney.

**Case study:**

Male, 58y, went through a routine abdominal ultrasound which showed a 5cm left kidney nodule. His MRI pointed a total of 4 nodules on his left kidney. The aspect suggested a papillary cancer due to high cellularity and low vascularization. The patient was submitted to partial nephrectomy under ischemia to remove the two largest tumors (inferior pole) and a resection without clamping of the other two ipsilateral tumors.

**Result:**

We performed the surgery in 2 stages. In the first one, we approached the 2 tumors located on the inferior pole inducing warm ischemia, whereas in the second stage we resected the 2 remaining tumors using the technique without clamping. The surgery lasted 220 minutes, with 800mL of blood loss, not requiring blood transfusion. Ischemia time was 35 minutes. The histopathological analysis confirmed that the 4 tumors were papillary cancer, with free margins.

**Conclusion:**

NSS can be performed and should be tried in patients with multiple kidney tumors, preferably through laparoscopy or assisted by robot. It can be made either using or not clamping of the pedicle, depending on the RENAL score.

## ARTICLE INFO

Available at: http://www.intbrazjurol.com.br/video-section/20150648-curcio_et_al/

Int Braz J Urol. 2017; 43 (Video #9): 567-7

